# Combined use of multimodal techniques for the resection of glioblastoma involving corpus callosum

**DOI:** 10.1007/s00701-021-05008-6

**Published:** 2021-10-12

**Authors:** Meng Cui, Hewen Chen, Guochen Sun, Jialin Liu, Meng Zhang, Hepu Lin, Caihong Sun, Xiaodong Ma

**Affiliations:** 1 Medical School of Chinese PLA, Beijing, 100853 China; 2 Department of Neurosurgery, the First Medical Center, Chinese PLA General Hospital, Beijing, 100853 China; 3 Department of Neurosurgery, the Seventh Medical Center, Chinese PLA General Hospital, Beijing, 100700 China

**Keywords:** Glioblastoma, Corpus callosum, Neuronavigation, Intraoperative magnetic resonance, Intraoperative neuromonitoring, Survival

## Abstract

**Purpose:**

To compare the multimodal techniques (including neuronavigation, intraoperative MRI [iMRI], and neuromonitoring [IONM]) and conventional approach (only guided by neuronavigation) in removing glioblastoma (GBM) with corpus callosum (CC) involvement (ccGBM), their effectiveness and safety were analyzed and compared.

**Methods:**

Electronic medical records were retrospectively reviewed for ccGBM cases treated in our hospital between January 2016 and July 2020. Patient demographics, tumor characteristics, clinical outcomes, extent of resection (EOR), progression-free survival (PFS), and overall survival (OS) were obtained and compared between the multimodal group (used multimodal techniques) and the conventional group (only used neuronavigation). Both groups only included patients that had maximal safe resection (not biopsy). Postoperative radiochemotherapy was also performed or not. Univariate and multivariate analyses were performed to identify significant prognostic factors and optimal EOR threshold.

**Results:**

Finally 56 cases of the multimodal group and 21 cases of the conventional group were included. The multimodal group achieved a higher median EOR (100% versus 96.1%, *P* = 0.036) and gross total resection rate (60.7% versus 33.3%, *P* = 0.032) and a lower rate of permanent motor deficits (5.4% versus 23.8%, *P* = 0.052) than the conventional approach. The multimodal group had the longer median PFS (10.9 versus 7.0 months, *P* = 0.023) and OS (16.1 versus 11.6 months, *P* = 0.044) than the conventional group. Postoperative language and cognitive function were similar between the two groups. In multivariate analysis, a higher EOR, radiotherapy, and longer cycles of temozolomide chemotherapy were positive prognostic factors for survival of ccGBM. An optimal EOR threshold of 92% was found to significantly benefit the PFS (HR = 0.51, *P* = 0.036) and OS (HR = 0.49, *P* = 0.025) of ccGBM.

**Conclusion:**

Combined use of multimodal techniques can optimize the safe removal of ccGBM. Aggressive resection of EOR > 92% using multimodal techniques combined with postoperative radiochemotherapy should be suggested for ccGBM.

**Supplementary Information:**

The online version contains supplementary material available at 10.1007/s00701-021-05008-6.

## Introduction

Gliomas are the most common primary intracranial tumor, representing 81% of malignant brain tumors. Although relatively rare, they cause significant mortality and morbidity. Glioblastoma (GBM) is the most invasive type of gliomas, with overall incidence rates ranging from 0.59 to 3.69 per 100,000 persons [32]. GBM is very invasive, typically infiltrating along white matter tracts [17]. As the largest interhemispheric fiber bundle in the human brain, the corpus callosum (CC) is frequently invaded by GBM. The GBM with CC involvement (ccGBM) can be classified as two types. For the first type, the lesion only invades one side of the hemisphere and the CC (non-butterfly ccGBM). The other type is known as butterfly GBM (bGBM), which involves the corpus callosum and both cerebral hemispheres [20, 23, 26, 30].

The ccGBM has a poorer prognosis than the GBM without CC involvement and is associated with incomplete resection or residual tumor after surgery [4, 16, 25, 28]. Some previous studies demonstrated that surgical resection prolonged the median overall survival (OS) of ccGBM and bGBM [3, 4, 6, 31]. Other studies argued that the median OS of the surgery group was similar to that of the biopsy group; furthermore, surgery cannot improve the quality of life or the Karnofsky performance score (KPS) [13, 20]. Therefore, a conventional idea is that it is best to perform biopsy to establish the diagnosis and to palliate these patients with noninvasive therapies (chemoradiotherapy or palliative care alone) [1, 10].

To date, the management of ccGBM is still controversial, and the optimal extent of resection (EOR) for the improvement of survival varies among studies. In recent years, we used multimodal techniques, including neuronavigation, intraoperative neuromonitoring (IONM), and intraoperative MRI (iMRI), to remove ccGBM. When all these elements were combined, we expected to achieve the optimal EOR and survival of ccGBM without severe complications and neurological deficits. In this retrospective study, by analyzing the clinical data of patients at our institution, we aimed to validate the use of advanced multimodal techniques can achieve the goal of maximal safe resection without causing more neurological deficits and prolong the survival in patients with ccGBM. It was also evaluated that bGBM may have similar good outcome and prognosis as non-butterfly ccGBM by using multimodal techniques in surgery. We also aimed to identify factors that affect the survival of ccGBM and the optimal threshold of EOR for ccGBM.

## Methods

### Patient selection

Retrospective clinical data of 484 patients with GBM were obtained from electronic medical records (EMRs) in the Department of Neurosurgery at our hospital between January 2016 and July 2020. The study was approved by our institutional ethics committee, and written informed consent for surgery was previously provided by all the patients or their relatives. The EMRs were reviewed to identify patients with ccGBM who met the following criteria: (1) resection in our department; (2) histopathology and molecular pathology confirmed as GBM (WHO grade IV) according to the 2016 WHO Classification of Tumors of the Central Nervous System; (3) tumor invading the CC partially or totally on preoperative MRI; (4) treatment using multimodal techniques of neuronavigation, IONM, and iMRI, or not; and (5) molecular pathology. All the surgeries were performed by XM. The exclusion criteria were as follows: (1) isolated lesion of the CC, (2) had only biopsy, (3) other isolated lesions existed other than ccGBM, (4) diffuse midline glioma with H3K27M mutation, and (5) patients lost to follow-up.

### Patient groupings

All patients with glioma were performed surgery by using neuronavigation at our institution from 2008. The patients with ccGBM were divided into the multimodal group (combined use of neuronavigation, iMRI, and/or IONM) and the conventional group (only used neuronavigation). The bGBM group (defined as tumor involved the CC and both cerebral hemispheres) and non-butterfly ccGBM group (defined as lesion only invaded one side of the hemisphere and the CC) were also evaluated to compare their clinical outcomes and survival.

### General clinical variables

General clinical variables and outcome variables were extracted by reviewing the EMRs from patients. Preoperative variables included age, sex, symptoms, muscle strength grading by the Medical Research Council (MRC) scale, aphasia quotient (AQ) by Western Aphasia Battery testing (values ≥ 93.8 and < 93.8 were defined as normal and aphasia respectively), cognitive function evaluated by the Montreal Cognitive Assessment (MoCA) scale, and KPS to evaluate a patient’s general functional status. Tumor-related variables included tumor location, recurrent or not, tumor volume, and molecular pathological findings (IDH 1/2 mutation status and methylation status of the MGMT promoter). Treatment-related variables included radiotherapy and cycles of temozolomide (TMZ) chemotherapy.

### Outcome variables

Outcome variables were generated by the follow-up data. They included length of hospital stay, surgery-related complications, EOR, muscle strength, AQ, MoCA score, and KPS at different time points, preservation rates of neurological function, progression-free survival (PFS), and overall survival (OS). If the tumor invaded or was located close to the eloquent area of neurological function (motor, language, and cognition), this patient was defined as a related case of neurological function needed be preserved. The preservation rate was defined as cases of finally functional preservation (function did not deteriorate or improved)/related cases.

### Image acquisition

Conventional MRI was performed for all patients on a 1.5-T scanner (Siemens Espree, Erlangen, Germany). The scanning sequences included T1-weighted, 3-D magnetization-prepared rapid-acquisition gradient echo (MPRAGE) sequences, T2-weighted images, T2 fluid-attenuated inversion recovery (FLAIR) images, and postcontrast 3D T1-weighted (T1C) images. If the tumor invaded or was close to eloquent cortices and tracts, blood oxygen level–dependent (BOLD) functional MRI (fMRI) and diffusion tensor imaging (DTI) were performed to detect eloquent cortices and white matter fibers. The iMRI or MRI within 48 h after surgery was performed to assess the residual tumor and EOR.

### Volumetric analysis

The digital imaging and communications in medicine (DICOM) data of all MRI sequences were transferred to iPlan software 2.6 (Brainlab, Feldkirchen, Germany). A region of interest (ROI) was manually drawn using an object creation module in the program by a board-certified neuroradiologist with 8 years of experience. The tumor boundary was delineated on T1C sequences of MRI. Pre- and postoperative tumor volumes (cubic centimeters [cm^3^]) were calculated automatically by the software on the basis of the tumor tissue seen on T1C images. The pre- and postoperative volumes of CC invaded by the tumor were also calculated by the software. The EOR was defined as (preoperative tumor volume − postoperative residual tumor volume)/preoperative tumor volume × 100. The EOR was divided into 4 categories: gross total resection (GTR: 100%), near total resection (NTR: 90–99%), subtotal resection (STR: 85–90%), and partial resection (PR < 85%) [1].

### Surgical plan

BOLD-fMRI data were used to locate the eloquent cortex, which included the motor cortex, the visual cortex, and the Broca and Wernicke areas. DTI data were used to reconstruct the pyramidal tract, the optic radiation, and the arcuate fasciculus through the fiber tracking module of iPlan 3.0 (Brainlab). Through the preoperative plan of 3D-visualized tumor and functional structures, the surgeon optimized the surgical approach, the incision, and the craniotomy. According to the extent of the tumor, uni- or bilateral craniotomy was performed to expose the tumor area maximally in a minimally invasive way.

### Surgical process

All surgeries were performed using general anesthesia and neuronavigation in an iMRI-compatible operating room (OR). After the bone flap was removed and the dura was opened, the surgeon started to remove the tumor. For bGBM invading bilateral brain lobes, to protect the functional cortex and tracts as much as possible, the surgeon started to remove the tumor via transcortical approach from the side that had maximal tumor burden guided by neuronavigation both on the screen and under the microscope. If both sides of the brain had similar tumor volume, we chose the nondominant hemisphere to perform corticectomy to make a resection corridor. When the ipsilateral tumor was completely removed, we exposed and resected the contralateral tumor through the longitudinal fissure. Most tumors can be removed below the cerebral falx. Sometimes, we resected part of the falx to provide a better view of the contralateral tumor. Furthermore, an angled endoscope can be used to explore the contralateral tumor. Because we created a corridor on the medial surface of the brain through the longitudinal fissure, the contralateral tumor (even distal tumor) can be exposed clearly and removed, and so can the contralateral cortices be preserved. If the cingulate gyrus had been invaded by the tumor, it will be sacrificed to achieve maximal safe resection. If it was not invaded, we exposed and removed the contralateral tumor above and below the cingulate gyrus; finally, the resection can converge and the cingulate gyrus can be completely preserved. In addition, MR angiography and venography can be fused with other sequences of MRI and displayed in neuronavigation, so that the location of the tumor and important arteries and veins can be shown visually to the surgeon.

For example, we cut through the cortex from the unilateral prefrontal lobe to expose the tumor, then removed the bGBM invading the bilateral frontal lobes and the genu of the CC, while avoiding the pyramidal and arcuate tracts behind the tumor in order to protect the motor and language functions. To expose the contralateral inferior tumor, we resected the tumor that involved the genu of the CC through the longitudinal fissure (removed the septum pellucidum if it was invaded) to create a corridor below the cingulate. To expose the contralateral upper and anterior tumor, we removed some subcortical parts of the contralateral frontal lobe and the falx (if necessary) to create a corridor above the cingulate. We did not remove the falx in most situations so as not to cause accidental injury to contralateral arteries. The tumor was removed under the pia mater between the windows of the branches of the anterior cerebral artery in order to preserve these vessels. These two resection corridors can converge to achieve a gross resection of the contralateral tumor. Finally, the resection cavities of the bilateral brain lobes can converge. Similarly for the bGBM invading the bilateral parietal–occipital lobes and splenium of the CC, we performed corticectomy on the unilateral non-eloquent cortex (usually through the parietal–occipital sulcus) to expose the tumor from above; thus, we can avoid the optic radiation below and behind the tumor to protect the visual function. The surgical process was similar with that mentioned above, so we can protect the posterior cingulate as well. Moreover, the main deep veins such as the internal cerebral vein (below the CC) and Galen vein (behind the CC) should be protected carefully when the splenium of the CC is resected.

**Fig. 1:**
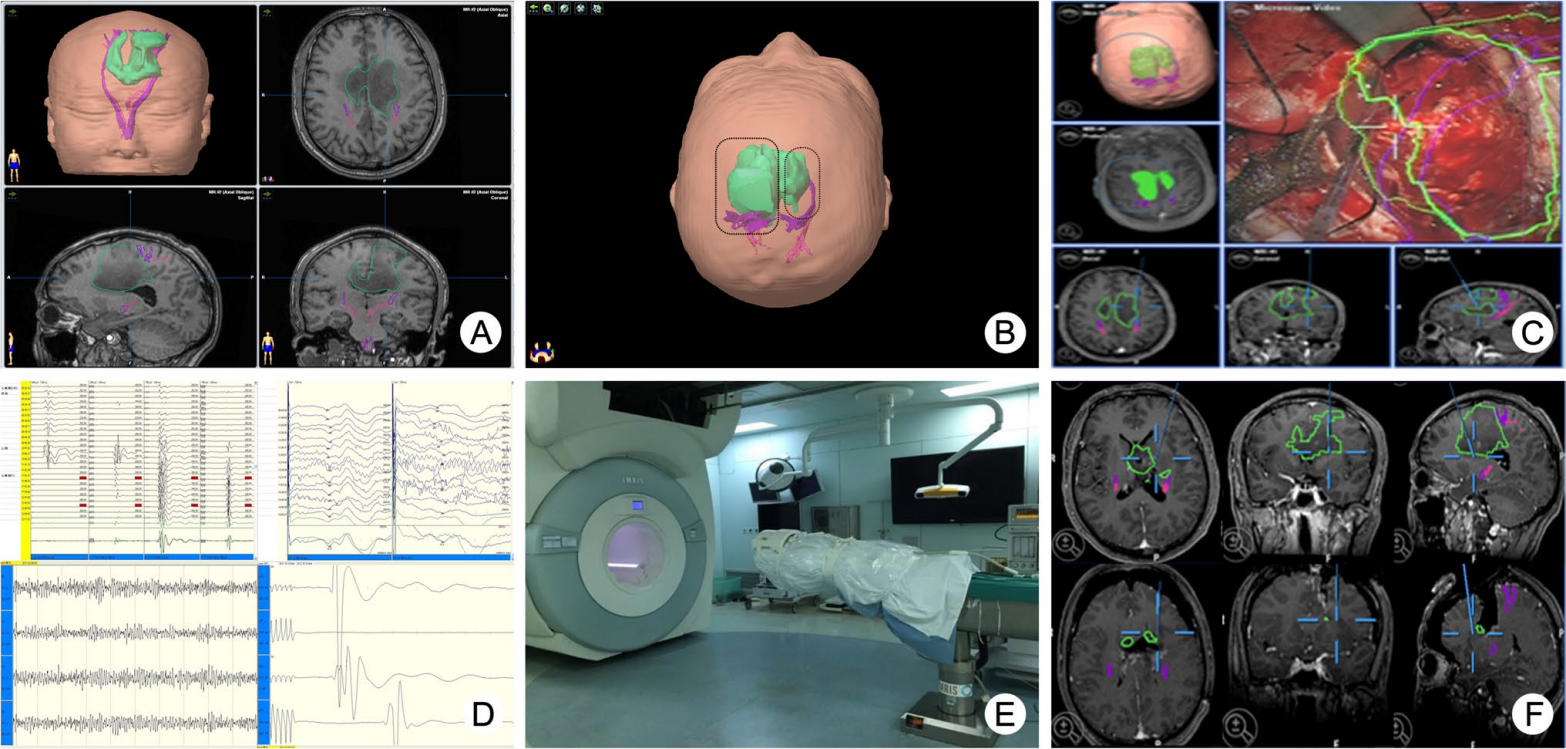
Resection procedure assisted by multimodal techniques. **A** Preoperative plan of neuronavigation designed by the surgeon, sensory tracts (pink), motor tracts (purple), and tumor (green). **B** Bilateral minimally invasive craniotomy was designed to avoid injury to the superior sagittal sinus. **C** Resection guided by neuronavigation on a screen and under a microscope. Tumor was started to be removed via corticectomy avoiding eloquent cortices from the side that had the maximal tumor burden. **D** Continuous MEP, SEP, EEG, and EMG monitoring, as well as their alterations during the resection process. **E** iMRI magnet moved to the OR for scanning. **F** The residual tumor was identified on iMRI images. According to the iMRI DICOM data, the neuronavigation was updated to perform further resection

If the tumor was located close to eloquent areas, IONM was also used (Endeavor CR system, Nicolet®, USA). Direct cortical and subcortical stimulations were performed using monopolar imaging to identify the functional cortex and tracts. Motor evoked potentials were continuously monitored by a neurophysiologist with 6 years of experience [22]. When the tumor was thought to have been completely removed by the surgeon, the surgical field was covered with sterile drapes. Then, the iMRI magnet was moved to the imaging position of the OR semiautomatically. During the iMRI scanning, the anesthesiologist followed the patient’s vital signs remotely through the monitor. If a residual tumor was identified on the T1C sequence of the iMRI, the DICOM data from the iMRI were imported into the iPlan software to update the surgical plan. Then, the surgeon continued to remove the residual tumor assisted by neuronavigation and IONM. During surgery, critical structures, such as the cingulum, basal ganglia, and anterior cerebral artery branches, can be displayed, and the surgeon can avoid injury to these structures that might cause postoperative complications (Fig. 1).

### Postoperative treatment and follow-up

According to the National Comprehensive Cancer Network (NCCN) guidelines, radiotherapy plus concomitant and adjuvant TMZ chemotherapy of ≥ 6 cycles were recommended for all patients with ccGBM [29, 36]. Regular MRI scans and follow-up were performed every 3 months for the patients.

### Statistical analysis

Statistical analysis was performed using SPSS 21.0 software (SPSS Inc., Chicago, IL, USA). The Shapiro–Wilk test was used to test the normality of the data. Student’s *t* and *χ*^2^ (or Fisher’s exact test) tests were used to compare continuous parametric and categorical variables between groups, respectively. The Mann–Whitney *U* test was used to compare continuous nonparametric variables, such as EOR, between groups. Survival curves were estimated by the Kaplan–Meier method and were compared by the log-rank test. Univariate and multivariate Cox proportional hazard models were used to identify significant prognostic factors of PFS and OS. To identify the threshold of EOR for ccGBM, serial multivariate analyses were performed in increments of 1% EOR. A *P* value < 0.05 was considered statistically significant.

## Results

**Fig. 2:**
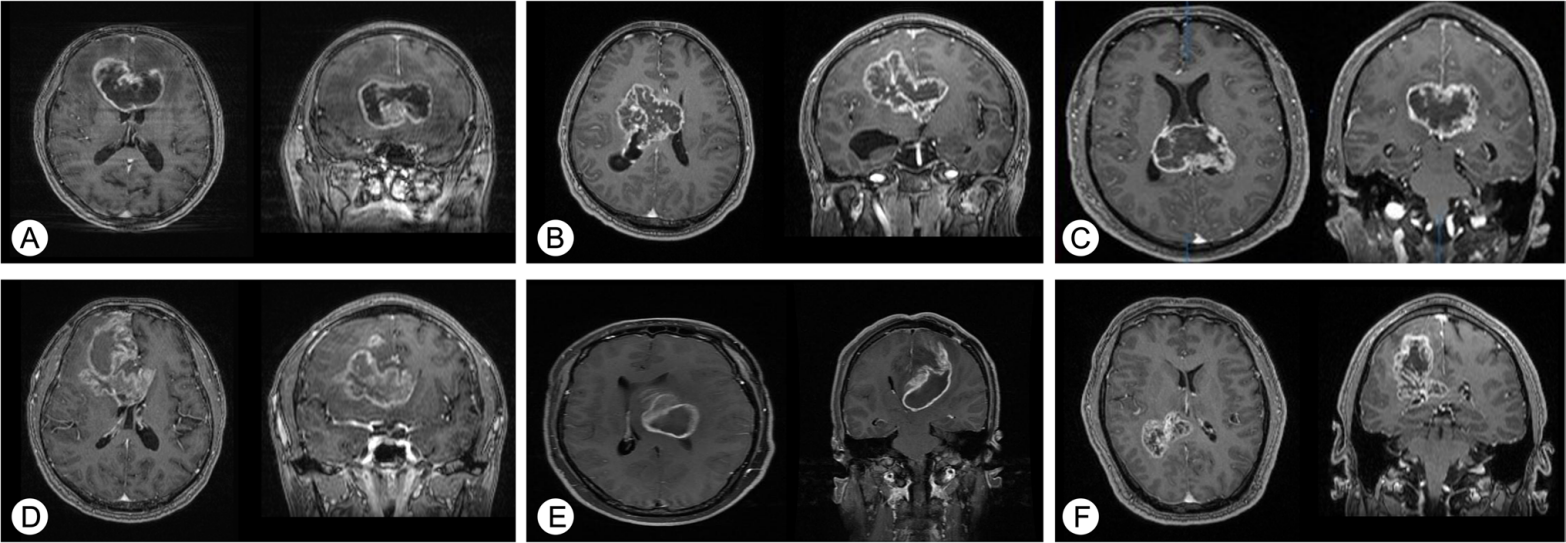
Characteristics of ccGBM that invaded different parts of the corpus callosum. All images originated from axial and coronal postcontrast 3D T1-weighted MRI. Genu invasion of the CC (**A**, **D**), body invasion of the CC (**B**, **E**), and splenium invasion of the CC (**C**, **F**). The upper column shows the bGBM (**A**–**C**); the lower column shows the non-butterfly ccGBM (**D**–**F**)

Among 484 GBM patients, 92 cases of ccGBM were identified, accounting for 19.0% of GBM. Five patients who underwent biopsy only, 6 patients who were lost to follow-up, and 4 patients who had isolated lesions other than ccGBM were excluded. Ultimately, 77 cases of ccGBM that underwent resection were included: 56 cases (27 bGBM and 29 non-butterfly ccGBM) were performed resection by using multimodal techniques (multimodal group) and 21 cases (9 bGBM and 12 non-butterfly ccGBM) were performed resection only guided by neuronavigation (conventional group). In addition, among all the ccGBM, 36 cases were bGBM, accounting for 7.4% of GBM, and 41 cases were non-butterfly ccGBM (Fig. 2).

### Clinical and tumor characteristics

The clinical and tumor characteristics of the two groups are summarized in Table 1. There were no significant differences in age, sex, muscle strength, AQ, MoCA score, symptoms, proportion of recurrent tumor, proportion of bGBM, location and volume of tumor, radio- and chemotherapy, status of IDH 1/2 mutation, or MGMT methylation between the multimodal and conventional groups.

**Table 1:** Baseline clinical and tumor characteristics between the multimodal and conventional groups

Variables	Multimodal group (*N* = 56)	Conventional group (*N* = 21)	*P* value
Age (years)^†^	49.4 ± 14.0	49.4 ± 10.5	0.987
Sex, *N* (%)			0.512
Male	30 (53.6)	13 (61.9)	
Female	26 (46.4)	8 (38.1)	
Muscle strength			
Related cases	39 (69.6)	11 (52.4)	0.157
Grades (0–5)^‡^	4.1 ± 1.3	4.3 ± 1.0	0.547
Language			
Related cases	17 (30.4)	6 (28.6)	0.879
AQ^‡^	94.6 ± 9.4	94.4 ± 9.8	0.696
Cognitive impairment			
Related cases	48 (85.7)	14 (66.7)	0.120
Median MoCA score (IQR)^‡^	27 (21–30)	27 (19.5–30)	0.790
Median preop. KPS (IQR)^‡^	70 (60–80)	80 (60–85)	0.515
Other symptoms, *N* (%)			
Headache	38 (67.9)	12 (57.1)	0.380
Seizure	7 (12.5)	2 (9.5)	1
Nausea/vomiting	12 (21.4)	5 (23.8)	1
Hypoesthesia	4 (7.1)	2 (9.5)	1
Vision defect	3 (5.4)	1 (4.8)	1
bGBM, *N* (%)	27 (48.2)	9 (42.9)	0.675
Non-butterfly ccGBM, *N* (%)	29 (51.8)	12 (57.1)	
Tumor location			
Frontal lobe	35 (62.5)	9 (42.9)	0.121
Frontal insular/temporal lobe	15 (26.8)	7 (33.3)	0.571
Parietal/parietooccipital lobe	6 (10.7)	5 (23.8)	0.273
Site of CC invasion, *N* (%)			
Genu	36 (64.3)	10 (47.6)	0.184
Body	6 (10.7)	1 (4.8)	0.716
Splenium	5 (8.9)	5 (23.8)	0.177
Genu and body	8 (14.3)	3 (14.3)	1
Splenium and body	1 (1.8)	2 (9.5)	0.179
Preop. total tumor vol (cm^3^)^‡^	59.33 ± 40.30	60.40 ± 27.24	0.354
CC invasion vol (cm^3^)^‡^	5.59 ± 3.60	7.07 ± 4.19	0.128
Ratio of CC invasion/total vol (%)^‡^	11.39 ± 6.65	12.64 ± 5.98	0.287
IDH 1/2 mutation, *N* (%)	15 (26.8)	9 (42.9)	0.175
MGMT methylation, *N* (%)	22 (39.3)	10 (47.6)	0.509
Radiotherapy, *N* (%)	41 (73.2)	16 (76.2)	0.791
Median TMZ cycles (range)^‡^	5.5 (0–31)	4 (0–24)	0.851

^†^Calculated by independent samples *t* test

^‡^Calculated by Mann–Whitney *U* test

### Outcomes and survival

**Fig. 3:**
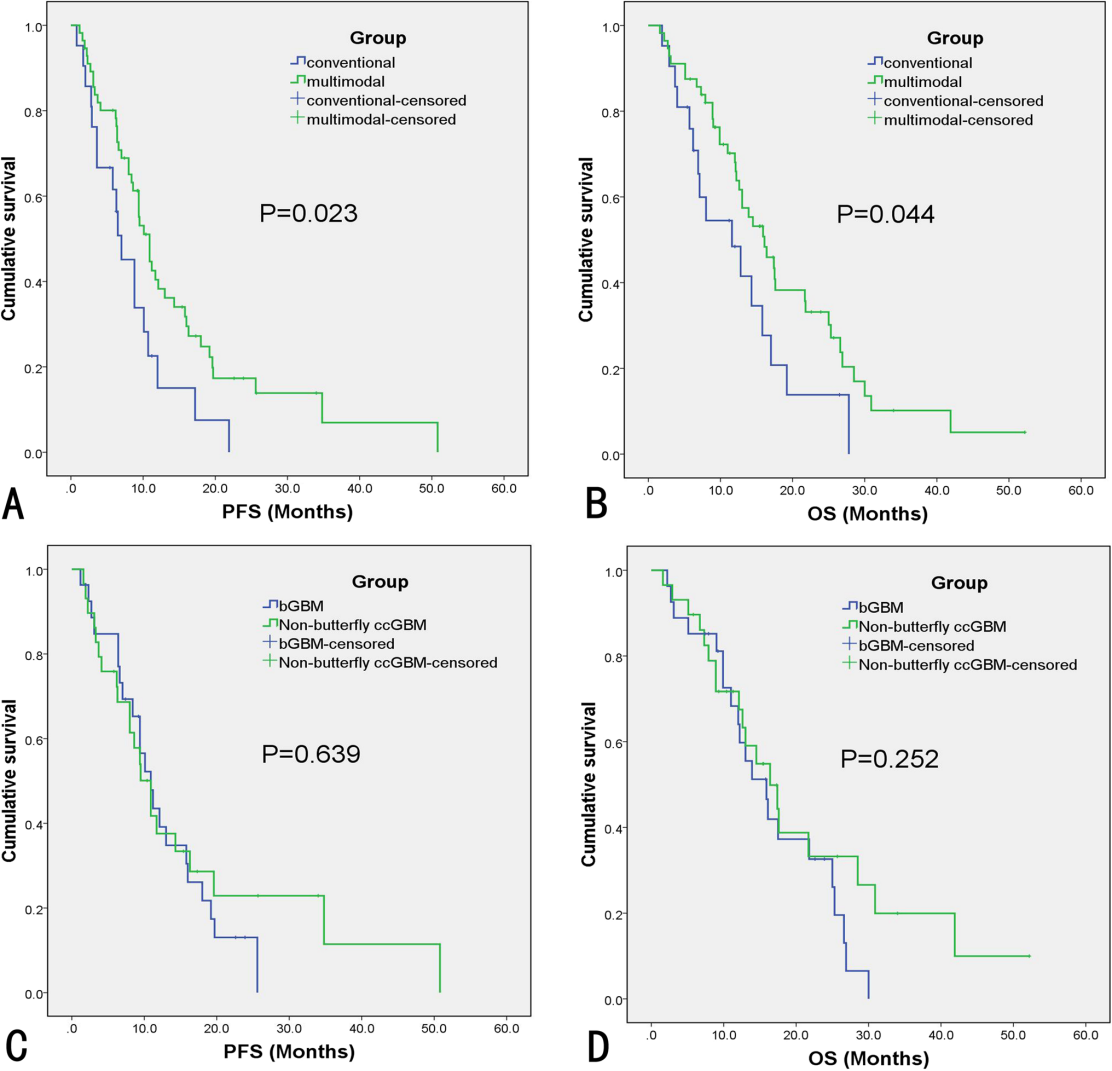
Kaplan–Meier survival curves of different groups. Multimodal group versus conventional group (**A**, **B**); bGBM versus non-butterfly ccGBM (**C**, **D**)

The multimodal group had a higher median EOR (100% [IQR: 96.39–100%] versus 96.1% [IQR: 87.71–100%], *P* = 0.036) than the conventional group. The multimodal group also had the higher rate of GTR (*P* = 0.032) than the conventional group. The muscle strength, AQ, MoCA score, and KPS were not different between the multimodal and conventional groups at different time points. However, the multimodal group had a higher preservation rate of motor function than the conventional group (92.3% versus 54.5%, *P* = 0.011). The multimodal group also had a lower incidence of permanent motor deficit than the conventional group (5.4% versus 23.8%, *P* = 0.052). The survival analysis demonstrated that the multimodal group had a longer median PFS (9.5 versus 7.0 months, *P* = 0.025) and OS (15.9 versus 11.6 months, *P* = 0.039) than the conventional group (Table 2). The survival curves are presented in Fig. 3.

**Table 2:** Outcomes of the multimodal group and the conventional group

Variables	Multimodal group (56)		Conventional group (21)		*P* value^*^
Length of hospital stay (IQR)^‡^	20 (15–25)		17 (14–22.5)		0.433
Surgery-related complications, *N* (%)	10 (17.9)		5 (23.8)		0.792
EOR, % (IQR)	100 (96.39–100)		96.10 (87.71–100)		**0.036**
Rate of GTR (%)	34 (60.7)		7 (33.3)		**0.032**
Rate of NTR (%)	16 (28.6)		7 (33.3)		0.411
Muscle strength^‡^					
Preoperative	4.1 ± 1.3	*P*^#^	4.3 ± 1.0	*P*^#^	0.547
At discharge	3.6 ± 1.8	0.362	3.5 ± 1.7	0.149	0.711
Postoperative 3 months	4.2 ± 1.2	0.772	3.4 ± 2.0	0.153	0.152
Preservation rate, *N* (%)^§^	36 (92.3)		6 (54.5)		**0.011**
Permanent deficits, *N* (%)^¶^	3 (5.4)		5 (23.8)		**0.052**
AQ^‡^					
Preoperative	94.6 ± 9.4	*P*^#^	94.4 ± 9.8	*P*^#^	0.696
At discharge	93.7 ± 10.0	0.429	87.4 ± 15.3	0.096	0.180
Postoperative 3 months	93.5 ± 9.7	0.513	88.7 ± 13.6	0.151	0.254
Preservation rate, *N* (%)^§^	14 (82.4)		3 (50.0)		0.279
Permanent deficits, *N* (%)^¶^	3 (5.4)		3 (14.3)		0.410
MoCA score^‡^					
Preoperative	27 (21–30)	*P*^#^	27 (19.5–30)	*P*^#^	0.790
At discharge	27 (18–30)	0.956	24 (16–30)	0.549	0.339
Postoperative 3 months	27 (18.25–30)	0.914	25 (16–30)	0.567	0.315
Preservation rate, *N* (%)^§^	44 (91.7)		10 (71.4)		0.125
Permanent deficits, *N* (%)^¶^	4 (7.1)		4 (19.0)		0.269
KPS (IQR)^‡^					
Preoperative	70 (60–80)	*P*^#^	80 (60–85)	*P*^#^	0.515
At discharge	80 (62.5–90)	0.080	70 (60–90)	0.908	0.605
Postoperative 3 months	70 (50–90)	0.922	80 (35–90)	0.939	0.867
Median PFS in months (95% CI)^‡^	10.9 (8.9 –12.9)		7.0 (4.5–9.5)		**0.023**
Median OS in months (95% CI)^‡^	16.1 (12.0–20.2)		11.6 (4.6–18.6)		**0.044**

Boldface type indicates statistical significance

^*^Comparison between the multimodal group and the conventional group

^#^Comparison between the preoperative and the postoperative neurological function

^‡^Calculated by Mann–Whitney *U* test

^§^Cases that did not have function deterioration at 3 months postoperatively divided by preoperative-related cases of tumors influencing the motor, language, or cognition

^¶^Cases that had permanent neurological deficits divided by total cases

### Findings of iMRI and postoperative MRI

**Fig. 4:**
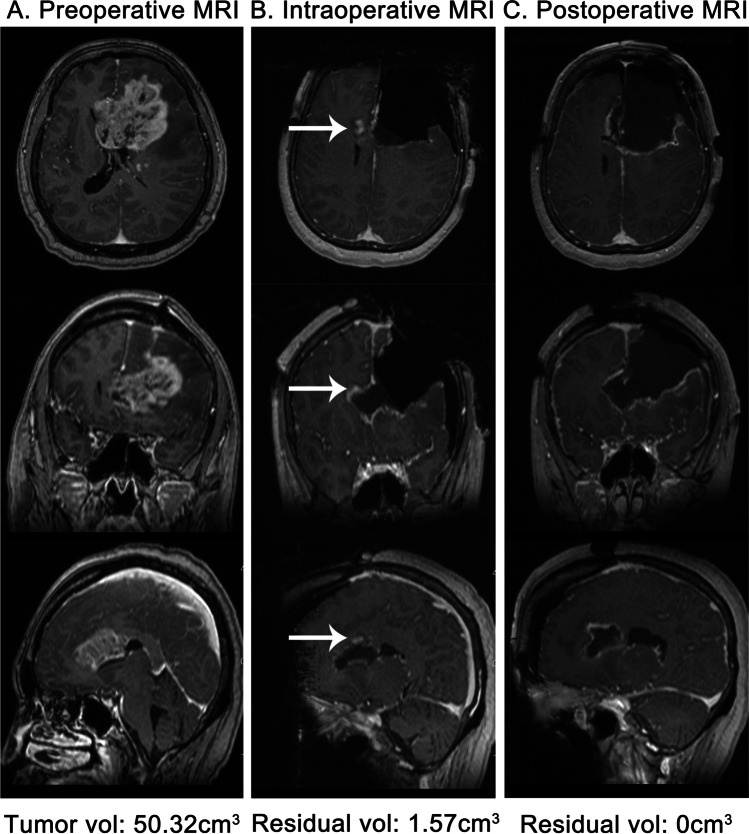
A case of using iMRI to increase the EOR for ccGBM. The patient was a 61-year-old woman who had a pathologic diagnosis of glioblastoma (WHO grade IV). The pre- (**A**), intra- (**B**), and postoperative (**C**) MRIs showed the residual tumor and further resection that increased the EOR from 96.88 to 100%. Volumetric measurements were automatically made by Brainlab. The arrow shows that the residual tumor was located in the contralateral corpus callosum

In the analysis of iMRI and postoperative MRI, when the tumor was removed from the side that had maximal tumor burden, the residual tumors were often located in the distal part of the contralateral brain lobe (16.1%) and the contralateral and bilateral corpus callosum (25%). In addition, there were always residual tumors in the deep part of the brain close to the basal ganglia and the eloquent area (12.5%). In the multimodal group, 13 patients had residual tumors on the first iMRI scan and then underwent more than one iMRI scan and further resection. The median EOR increased from 90.23 to 100% (*P* < 0.001). Five (38.5%) patients had an increased EOR of more than 10% (Fig. 4).

### Comparison of bGBM and non-butterfly ccGBM in the multimodal group

The clinical and tumor characteristics of bGBM (27 patients) and non-butterfly ccGBM (29 patients) are summarized in Table 3, which showed no differences existed between the two groups. By using the multimodal approach, the clinical outcomes, including the length of hospital stay, EOR, and postoperative KPS, were proved to be similar between bGBM and non-butterfly ccGBM. The survival analysis demonstrated the median PFS (10.9 versus 10.9 months, *P* = 0.639) and OS (15.9 versus 16.4 months, *P* = 0.252) were not different significantly.

**Table 3:** Comparison between the bGBM and non-butterfly ccGBM in the multimodal group

Variables	bGBM (*N* = 27)	Non-butterfly ccGBM (*N* = 29)	*P* value
Age (years)^†^	49.6 ± 15.2	49.2 ± 13.1	0.912
Sex, *N* (%)			0.058
Male	18 (66.7)	12 (41.4)	
Female	9 (33.3)	17 (58.6)	
Median preop. KPS (IQR)^‡^	70 (60–80)	70 (65–80)	0.762
Preop. total tumor vol (cm^3^)^‡^	58.81 ± 45.81	59.81 ± 35.22	0.749
CC invasion vol (cm^3^)^‡^	6.43 ± 4.12	4.80 ± 2.90	0.144
Median ratio of CC invasion/total vol, % (IQR)^‡^	11.5 (8.9–16.6)	9.5 (4.8–13.6)	0.093
*IDH 1/2* mutation, *N* (%)	5 (18.5)	10 (34.5)	0.178
*MGMT* methylation, *N* (%)	13 (48.1)	10 (34.5)	0.299
Radiotherapy, *N* (%)	20 (74.1)	21 (72.4)	0.889
Median TMZ cycles (range)^‡^	4 (0–21)	6 (0–31)	0.537
Outcomes			
Length of hospital stay (IQR)^‡^	21 (16–25)	17 (13.5–25.5)	0.221
EOR, % (IQR)^‡^	100 (91.59–100)	100 (96.48–100)	0.434
KPS at discharge^‡^	80 (60–90)	80 (65–90)	0.381
KPS at 3 months^‡^	70 (40–90)	80 (50–95)	0.072
Median PFS in months (95% CI)^‡^	10.9 (8.2–13.6)	10.9 (8.2–13.6)	0.639
Median OS in months (95% CI)^‡^	15.9 (11.3–20.5)	16.4 (10.5–22.3)	0.252

^†^Calculated by independent samples *t* test

^‡^Calculated by Mann–Whitney *U* test

### Prognosis analysis of ccGBM

Univariate analysis showed that age, multimodal approach, EOR, radiotherapy, TMZ cycles, KPS on 3 months postoperatively, IDH mutation, and MGMT methylation were significantly associated with the PFS and OS of ccGBM (*P* < 0.05) (Table 4).

**Table 4:** Prognostic factors of ccGBM by univariate analysis

Multivariate analysis	Age	Multimodal approach	EOR	Radiotherapy	TMZ cycles	KPS on 3 months	IDH mutation	MGMT methylation
HR for PFS (95% CI)	1.02 (1.01–1.04)	0.53 (0.30–0.93)	0.94 (0.91–0.98)	0.27 (0.15–0.48)	0.88 (0.83–0.95)	0.99 (0.98–0.99)	0.48 (0.27–0.87)	0.59 (0.35–0.99)
*P* value	0.051	0.027	0.004	< 0.001	< 0.001	< 0.001	0.015	0.048
HR for OS (95% CI)	1.02 (1.00–1.05)	0.55 (0.30–0.99)	0.94 (0.90–0.98)	0.24 (0.13–0.42)	0.84 (0.77–0.91)	0.98 (0.98–0.99)	0.46 (0.24–0.88)	0.57 (0.33–1.00)
*P* value	0.029	0.048	0.004	< 0.001	< 0.001	< 0.001	0.018	0.048

Because the multimodal approach in surgery influenced survival by increasing EOR and KPS, multivariate analysis did not include this factor. The results showed that age, KPS on 3 months postoperatively, IDH mutation, and MGMT methylation were not correlated with the PFS and OS (*P* > 0.05) because of our comprehensive treatment (Table 5). The EOR, radiotherapy, and TMZ cycles were positively correlated with the PFS and OS. The higher EOR was associated with the longer PFS (HR = 0.94, *P* = 0.012) and OS (HR = 0.94, *P* = 0.009). Patients performed radiotherapy had longer PFS (HR = 0.44, *P* = 0.012) and OS (HR = 0.43, *P* = 0.011) than those without radiotherapy. More TMZ cycles meant longer PFS (HR = 0.93, *P* = 0.045) and OS (HR = 0.87, *P* = 0.005) as well. When all significant factors identified by univariate analysis are combined, the 92% threshold of EOR at least can prolong both the PFS (HR = 0.51, *P* = 0.036) and OS (HR = 0.49, *P* = 0.025) significantly (Table 6).

**Table 5:** Prognostic factors of ccGBM by multivariate analysis

Multivariate analysis	Age	EOR	Radiotherapy	TMZ cycles	KPS on 3 months	IDH mutation	MGMT methylation
HR for PFS (95% CI)	1.00 (0.98–1.03)	0.94 (0.90–0.99)	0.44 (0.23–0.83)	0.93 (0.86–0.99)	1.00 (0.99–1.01)	0.73 (0.36–1.48)	0.80 (0.45–1.43)
*P* value	0.745	**0.012**	**0.012**	**0.045**	0.402	0.381	0.451
HR for OS (95% CI)	1.01 (0.98–1.03)	0.94 (0.90–0.99)	0.43 (0.23–0.83)	0.87 (0.80–0.96)	1.00 (0.98–1.01)	0.94 (0.45–2.00)	0.67 (0.36–1.23)
*P* value	0.689	**0.009**	**0.011**	**0.005**	0.302	0.865	0.193

Boldface type indicates statistical significance

**Table 6:** EOR threshold identified by multivariate analysis in increments of 1% EOR

Multivariate Analysis	EOR (92%)	EOR (91%)	EOR (90%)
HR for PFS (95% CI)	0.51 (0.28–0.96)	0.55 (0.28–1.07)	0.61 (0.30–1.25)
*P* value	**0.036**	0.079	0.179
HR for OS (95% CI)	0.49 (0.26–0.91)	0.47 (0.24–0.93)	0.53 (0.25–1.11)
*P* value	**0.025**	**0.029**	0.091

Boldface type indicates statistical significance

## Discussion

Maximal safe resection combined with radiotherapy and chemotherapy is the current standardized approach for gliomas. Maximizing the extent of bulk tumor removal is the goal of glioma resection, which can improve the outcomes of patients, including PFS and OS [11, 34]. CC involvement is regarded as a poor prognostic factor in GBM [18, 25]. The ccGBM has been thought to be a more diffuse type of glioma that cannot be completely removed. The percentage of ccGBM in GBM ranged from 14.9 to 33.3% in previous studies [4, 25, 28], and the percentage of bGBM in GBM ranged from 2.0 to 14.3% [3, 6, 13, 16, 31]. In our cohort, ccGBM and bGBM accounted for 19.0% and 7.4% of GBM respectively, which were both within the range reported in previous studies. Recently, multimodal approaches, including neuronavigation, IONM, and iMRI, have made maximal safe resection of these lesions possible [19]. In this retrospective study, we used multimodal techniques to remove ccGBM and obtained satisfactory outcomes.

The preoperative clinical and tumor characteristics were not different between the multimodal group and the conventional group that only used neuronavigation, which demonstrated a good balance between the groups. The combination of multimodal techniques achieved a higher EOR than the conventional surgery without causing more complications like hemorrhage, ischemia, severe edema, etc. Its rates of GTR (EOR: 100%) and NTR (EOR: 90–99%) reached 60.7% and 28.6% respectively. Meanwhile, compared to the preoperative and conventional group’s neurological functional status, the multimodal approach both achieved the preservation of neurological function postoperatively, including the motor, language, and cognitive function. The resection using multimodal techniques can significantly prolong the PFS and OS of ccGBM than the conventional resection. Additionally, the bGBM had similar outcomes and survival with non-butterfly ccGBM after resection in the multimodal group. It demonstrated the multimodal approach can benefit both types of ccGBM, especially the bGBM. It was also because we used the proper resection approach to preserve functional cortices and tracts as well as the cingulate gyrus. The hemisphere that had maximal tumor burden or the nondominant hemisphere (if both hemispheres had similar tumor burdens) was chosen to be performed corticectomy to make a resection corridor. The contralateral tumor was removed through the longitudinal fissure so that the cortices of the contralateral brain lobe can be preserved as much as possible. The tumor was removed above and below the cingulate so as to preserve it. The injury of the frontal cortices may cause damage to cognitive function such as memory, comprehension, and calculation [12]. The injury of the cingulate may cause personality changes and abulia [33]. So the preservation of these structures by our proper surgical approach can achieve the protection of some advanced brain functions. Assisted by multimodal techniques, the eloquent cortices and tracts can be protected as well. Finally, we achieved the maximal safe resection of bGBM. Their survival and functional outcomes were similar with those of non-butterfly ccGBM.

Prognosis analysis of ccGBM showed that the multimodal approach was an important factor that improved the PFS (HR = 0.53, *P* = 0.027) and OS (HR = 0.55, *P* = 0.048) of ccGBM. The increment of EOR can also prolong the PFS and OS. If the EOR was increased to more than 92%, the PFS (HR = 0.51, *P* = 0.036) and OS (HR = 0.49, *P* = 0.025) can be prolonged significantly. Thus, the EOR must reach 92% as a reference threshold when removing the ccGBM. In this cohort, resection assisted by multimodal techniques resulted in 89.3% patients achieving an EOR of more than 90%. Postoperative radiotherapy and more cycles of TMZ chemotherapy both benefitted the survival of ccGBM patients. Although the age, IDH mutation, and MGMT methylation significantly influenced the survival of ccGBM in univariate analysis, they were proved to be not associated with the survival in multivariate analysis. Thus, multimodal technique–assisted surgery followed by postoperative radiotherapy and long cycles of TMZ chemotherapy can be suggested to manage all patients with ccGBM.

Some retrospective studies demonstrated that resection can benefit the overall survival of ccGBM patients. Only Dziurzynski (2012) reported that resection did not correlate with the survival (*P* = 0.14). Among these studies, five studies only reported the bGBM; other studies also included the non-butterfly ccGBM (Table 7). The median EOR of our study was higher than those of most previous studies. One study (Dziurzynski et al., 2012) reported a median EOR of 100%, but its samples were only 11 and it had five patients of EOR less than 65%. The rate (60.7%) of GTR of our study was higher than those of most previous studies. Furthermore, 89.3% of patients achieved an EOR that was more than 90% in our study; this rate was higher than that of most previous studies, while they were lower than those of Forster et al. (2020) and Burks et al. (2016) respectively. We thought the reason was that Forster et al. (2020) excluded the glioma that spread into eloquent areas (such as the basal ganglia) and Burks et al. (2016) also included low-grade glioma. The median OS of previous studies ranged from 7 to 15 months [1, 3, 4, 6, 13, 14, 16, 31]. Only Chen (2015) and Franco (2020) reported a median PFS of 6.2 and 4.2 months respectively. Because of the use of a multimodal approach, this study achieved longer median PFS and OS both for bGBM (10.9 and 15.9 months) and non-butterfly ccGBM (10.9 and 16.4 months) than previous studies. Postoperative complications, such as hemorrhage, ischemia, infection, and severe edema, were controlled at a lower incidence (17.9%) than those of previous studies. Sanai et al. (2011) reported that an EOR as low as 78% may provide a survival benefit for GBM. But Sanai et al. (2011) also reported the stepwise improvement in OS even in the 95–100% EOR range [34]. An influential study of Lacroix et al. (2001) reported an EOR threshold of 98% for GBM which was associated with significant survival advantage [24]. Thus, even if an EOR threshold of 78% was achieved, a much higher EOR for GBM should be pursued as far as possible. The optimal EOR thresholds for ccGBM ranged from 65 to 86% in previous studies. Our result supported this view and reported a much higher EOR threshold (92%) for ccGBM than previous studies, which meant a much higher EOR and better outcomes can be pursued for this type of aggressive and incurable tumor. There were 80.4% of patients who achieved an EOR of more than 92% by using multimodal techniques in our study.

**Table 7:** Summary of studies on patients with ccGBM

Study	Patients of surgery (*N*)	Included patients	EOR, median (range) or mean ± SEM	100% GTR (%)	90–99% NTR (%)	EOR threshold	Median overall survival (months)	Overall complication rate
Franco et al., 2020	25	ccGBM	> 95% (*N* = 8 [32%]), < 95% (*N* = 17 [68%])	/	/	/	8.6	32.0%
Forster et al., 2020	17	ccGBM (WHO II = 1, WHO III = 3)	/	15 (71.4)	6 (28.6)	/	12.6	/
Dayani et al., 2018	14	Only bGBM	83.0% (44.2–100%)	/	/	86%	14.1	28.6%
Opoku-Darko et al., 2017	9	Only bGBM	> 98% (*N* = 5), < 98% (*N* = 4)	/	/		7.8	22.2%
Burks et al., 2016	21	bGBM (WHO II = 13, WHO = 6)	/	33 (82.5%)	3 (7.5%)	/	15.0	22.5%
Chen et al., 2015	18	ccGBM (WHO III = 4)	> 95% (*N* = 2), 75–95% (*N* = 7), < 75% (*N* = 10)	/	/	85%	12.5	/
Chaichana et al., 2014	29	Only bGBM	61.4 ± 4.9%	/	/	65%	7.0	/
Dziurzynski et al., 2012	11	Only bGBM	100% (24.7–100%)	6 (54.5)	0	/	8.8	/
Present study	56	ccGBM	100% (79.3–100%)	34 (60.7)	16 (28.6)	92%	16.1	17.9%
		bGBM (*N* = 27)	100% (91.59–100%)	15 (55.6)	8 (29.6)		15.9	22.2%

The use of iMRI continues to increase in neurosurgery as a tool to provide maximal EOR. No studies have evaluated the benefit of iMRI on the EOR of ccGBM. However, some studies have reported the influence of iMRI on all gliomas. A retrospective review of 42 glioma resections utilizing iMRI demonstrated that further resection was performed in 40.5% of cases with an increase in the mean EOR from 56 to 67% after iMRI [27]. Additionally, another study demonstrated that EOR was increased by an average of 10% with the use of iMRI [37]. Our data supported these findings. Thirteen patients with residual tumors on the first iMRI scan was performed multiple iMRI and further resection; the median EOR increased from 90.23 to 100%. The residual tumors were often located in the contralateral brain lobe, the corpus callosum, and the deep part of the brain close to the basal ganglia and the pyramidal tract, which reminded the surgeon to remove the tumor in these areas using multiple iMRIs more aggressively and meticulously to maximize the resection safely. In addition, the iMRI can help the surgeon make decisions during the surgery to balance EOR and neurological functions.

When the ccGBM is located in close proximity to eloquent structures, neurological function should be carefully protected in parallel with the attempt to increase EOR. Awake craniotomy with intraoperative neuromonitoring is the gold standard method of mapping motor, sensory, language, and visual cortices and white matter tracts [8, 15, 19]. In this study, neuronavigation and IONM were used, although awake surgery was not performed. By performing resection assisted by the multimodal approach, the incidences of permanent motor and language deficits were both reduced to 5.4%, which was similar to the incidence of previous studies (3.5–6.5%) [2, 8, 9, 21]. Some previous studies reported the incidence (7.1% in Dayani et al. (2018) and 13.8% in Chaichana et al. (2014)) of permanent motor and language deficits in ccGBM resection, which were lower than those of our study. In addition, we performed bilateral craniotomy for bGBM, removed the tumor from one side that had the larger tumor burden, and then removed the contralateral tumor through a longitudinal fissure by a subcortical approach in an attempt to protect the contralateral cortical and subcortical function as much as possible. This method allowed the protection incidence of cognitive function to reach 91.4%.

To the authors’ knowledge, this study is the largest series utilizing a multimodal approach guiding ccGBM resection. The results should be validated with further prospective studies. Further research should concentrate on the different treatment strategies for ccGBM invading different corpus callosum parts (genu, body, and splenium). The ccGBM invading the body or splenium of the corpus callosum is close to eloquent areas, and awake surgery combined with the present multimodal approach described in this study should be suggested and researched. For ccGBM invading the genu of the corpus callosum that is far from eloquent structures, the present multimodal approach in this study can be adequate. Given the paucity of literature regarding the resection of ccGBM, this study provides much-needed evidence to help guide treatment decision-making for patients with this disease. However, quality of life, especially advanced neurological function, cannot be assessed entirely by the KPS. Further research should assess the quality of life of patients with ccGBM in detail using assessment scales such as the Quality of Life Questionnaire Core 30 (QLQ-C30), Quality of Life Questionnaire-Brain Neoplasm 20 (QLQ-BN20), or other scales [7]. Therefore, the effect of the treatment on quality of life can be presented more entirely and accurately, which can guide surgeons to make the most suitable decisions for different patients. Previous studies showed a higher rate of *PDGFRA* alterations in gliomas of CC involvement [5, 35]. So additional molecular alterations of ccGBM should be considered in further studies to guide the treatment and prognosis precisely.

## Conclusions

This retrospective analysis reviewed the use of multimodal techniques to optimize the safe removal of ccGBM. This demonstrated the safety and effectiveness of this combined approach when resecting ccGBM including bGBM and non-butterfly ccGBM. The EOR can be increased significantly in parallel with the protection of neurological function. The EOR should reach the threshold of 92% to significantly benefit the survival of patients. Maximal safe resection assisted by a multimodal approach combined with postoperative radiochemotherapy should be suggested for all patients with ccGBM. Further prospective studies are urgently needed to find suitable treatment methods for different patients with ccGBM using other techniques, such as awake surgery, laser-induced interstitial therapy, tumor treatment fields, and other emerging approaches.

## Supplementary Information

Below is the link to the electronic supplementary material.

Supplementary file1 (XLSX 25 KB)

## Data Availability

The original contributions generated for this study are included in the article/Supplementary material. Further inquiries can be directed to the corresponding author. This retrospective study was approved by our institutional ethics committee and in accordance with the 1964 Helsinki declaration. Written informed consent for surgery was previously provided by all the patients or their relatives before surgery.
